# Updated View on the Relation of the Pineal Gland to Autism Spectrum Disorders

**DOI:** 10.3389/fendo.2019.00037

**Published:** 2019-02-05

**Authors:** Tal Shomrat, Nir Nesher

**Affiliations:** Ruppin Academic Center, Faculty of Marine Sciences, Michmoret, Israel

**Keywords:** autism, pineal gland, N, N-dimethyltryptamine (DMT), melatonin, neural plasticity

## Abstract

Identification of the biological features of autism is essential for designing an efficient treatment and for prevention of the disorder. Though the subject of extensive research, the neurophysiological features of autism remain unclear. One of the proposed biological causes of autism is malfunction of the pineal gland and deficiency of its principal hormone, melatonin. The main function of melatonin is to link and synchronize the body's homeostasis processes to the circadian and seasonal rhythms, and to regulate the sleep-wake cycle. Therefore, pineal dysfunction has been implicated based on the common observation of low melatonin levels and sleep disorders associated with autism. In this perspective, we highlight several recent findings that support the hypothesis of pineal gland/melatonin involvement in autism. Another common symptom of autism is abnormal neuroplasticity, such as cortical overgrowth and dendritic spine dysgenesis. Here, we synthesize recent information and speculate on the possibility that this abnormal neuroplasticity is caused by hyperactivity of endogenous N,N-dimethyltryptamine (DMT). The pineal gland was proposed as the source of DMT in the brain and therefore, our assumption is that besides melatonin deficiency, pineal dysfunction might also play a part in the development of autism through abnormal metabolism of DMT. We hope that this manuscript will encourage future research of the DMT hypothesis and reexamination of several observations that were previously attributed to other factors, to see if they could be related to pineal gland/melatonin malfunction. Such research could contribute to the development of autism treatment by exogenous melatonin and monitored light exposure.

## Introduction

Decades of intensive research have revealed a wide-range of physiological features (1, 2), genetic (3), and environmental effectors (4), which have been suggested to be the cause, trigger, or to enhance the risk of autism spectrum disorder (ASD). Yet, none of the hundreds of gene mutations or environmental conditions have been found to be consistently present across the wide spectrum of the heterogenic autistic population. One of the proposed biological causes of autism is malfunction of the pineal gland (5, 6).

The pineal gland is a minute neuroendocrine gland, located deep in the center of the brain, projecting from the posterior wall of the third ventricle. Its position on top of the third ventricle allows direct secretion to the cerebrospinal fluid (CSF), in addition to the main route of hormone secretion to the blood. Interestingly, an average excess of CSF volume in the subarachnoid space was detected in autistic infants (7). Increase in CSF volume could have an influence on the concentration and effectiveness of the components it delivers to the brain. Melatonin (N-acetyl-5-methoxytryptamine) is considered as the principal hormone produced by the pineal gland and its well-documented function is to link and synchronize the body's homeostasis processes to the circadian and seasonal rhythms, and regulate the sleep-wake cycle. Melatonin synthesis and secretion are mainly controlled by photosensory information that arrives from the suprachiasmatic nuclei (SCN). The SCN control of the pineal gland creates a strong circadian rhythm with high levels of melatonin secretion in the dark at night, and negligible levels during the day.

Melatonin functions through several receptors and mechanisms [for review, see, (8, 9)]. In short, the most well-characterized pathways, which are assumed to be the main targets of melatonin, are the membrane specific G protein-coupled melatonin receptors MT1 and MT2. These two principal receptors are expressed throughout the brain (9, 10). In addition, due to its lipophilic trait, melatonin is permeable to biological membranes and binds to intracellular proteins such as calmodulin, and directly and indirectly effects nuclear receptors like the receptor family RZR/ROR, which act as transcriptional activators and induce immunomodulatory effect. Moreover, recent studies proposed a putative binding site to quinone reductase II (MT3), a detoxification enzyme with metabolic and antioxidative effects (11).

Melatonin takes effect at early stages of embryo-fetal developmental. Actually, even before embryo development, melatonin affects oocyte quality, and possibly induces epigenetic modulation inside the oocyte (12, 13). During pregnancy the embryo depends on maternal melatonin since the pineal gland is activated only after birth. Maternal concentrations of melatonin increase after 24 weeks of gestation, with significantly higher levels after 32 weeks (14, 15). Melatonin receptors are widespread in the embryo and fetus from early stages, both in the nervous system, and in peripheral organs. Melatonin influences the internal rhythms of the offspring and has a direct developmental and neuroprotective effect on the fetal brain (13, 16). Besides the maternal source, melatonin is synthesized by the placenta which also express MT1 and MT2 receptors that mediate direct effects on placental maintenance (17). Vanecek (18) showed that in rat, the melatonin receptors in the pituitary gland decrease drastically postnatal, but maintain the same quantity in the hypothalamus. This finding implies different roles for melatonin during pregnancy and postnatal, and displays the dynamic distribution of melatonin receptors; as been found in the placenta during pregnancy (17) and for day/night expression in the brain (19). From the above it is clear that impairment of the melatonin pathway during pregnancy might increase risk of neurodevelopmental disorders, such as ASD (20).

The link between autism and pineal gland/melatonin malfunction was first recognized after observations revealing low concentrations of melatonin in individuals with autism (21). About 65% of ASD patients have less than half the average values of melatonin (22). Moreover, sleep disorder is one of the most common symptoms in ASD patients, with prevalence of 50–80%, compared to 9–50% in normal children (22, 23). Also, exogenous treatment with melatonin was found to be very beneficial in reducing the time until onset of sleep, and increasing sleep duration (6).

Genetically, autism-associated mutations have been found in genes encoding the key enzymes of melatonin synthesis, Aralkylamine N-acetyltransferase (AANAT), and Acetylserotonin O-methyltransferase (ASMT) (24, 25), as well as a reduced expression of the gene encoding AANAT in ASD (26). Ggene abnormalities have also been found in gene encoding the melatonin receptors MT1 and MT2. Though, a systematic review by Rossignol and Frye (6) concluded that the contribution of these genetic variations to melatonin deficiency are presented in only a small percentage of individuals with ASD.

Here, we highlight recent information that we suggest further supports the pineal gland/melatonin hypothesis. In addition to the link between melatonin and ASD, we propose new insights in light of the recent work by Calvin Ly et al. (27), speculating on the possibility that abnormal metabolism of endogenous N,N-dimethyltryptamine (DMT), possibly from the pineal gland (28), might cause the aberrant neuroplasticity and neural connectivity abnormalities observed in autistic patients (29, 30).

## Recent Findings Reveal a Possible Link Between Autism and Pineal Gland/Melatonin Malfunction

Braam et al. (31) included a Supplementary Table in their recent work that summarizes several observations that could be associated with melatonin deficiency in the autistic child or mother. Here we take some of their notes and expand the concept with our insights.

In addition to the chronobiological function, melatonin has two other putative roles. First, melatonin is considered to be one of the most efficient and vital endogenous antioxidants found in the brain and body tissues (32). Therefore, melatonin deficiency might cause neuronal damage induced by oxidative stress (33). Secondly, there is accumulating data indicating that melatonin is an immunomodulatory compound with immunostimulant or anti-inflammatory properties (34). Immune system abnormalities have been reported in children with autism (35) which might demonstrate an addition link between the pineal gland and ASD.

During embryonic and infant development, the pineal is the first gland to develop in the fetus, reaching its mature size at 2 years (36). However, melatonin production is not significant during the first 3 months of life. From the fourth month, the levels of nocturnal melatonin secretions steadily increase–reaching a peak at age three to five. This time scale has some degree of correlation with the age of autism diagnosis–after the first year and mainly between the ages of two to four, when the symptoms clearly present. Tauman et al. (37) reported an association between low melatonin excretion during the first prenatal weeks to delayed psychomotor development that first presented at 6 months of age; which correlates with the earliest time that ASD related symptoms might be evident (30). Aside from the above, reexamination of other observations from ASD could reveal novel correlations. **First**, melatonin synthesis is controlled by light absorbed by intrinsically photosensitive retinal ganglion cells (ipRGCs); these are specific light-sensitive cells that are separated from the principal visual photoreceptors. Malfunction in the ipRGCs area of the retina might provide an explanation for the association found between congenital visual impairment and the higher risk of developing autism (38, 39). This risk factor may the result of abnormal melatonin rhythms (40) and not due to melatonin deficiency since the average melatonin level is supposedly higher in blind subjects. **Additionally**, adults with ASD appear to be at significant risk for developing type 2 diabetes, coronary heart disease, and cancer (41, 42). These diseases and autism may share risk factors that are not directly linked to melatonin, such as obesity and genetic mutations. However, it has been suggested that melatonin is a protective factor against cancer (43) and coronary heart disease (44). There is also general agreement that melatonin metabolism has a link to type 2 diabetes (45), although the mechanism is still unclear (46).

**Lastly**, a set of recent studies have revealed that the prevalence rates of ASD are lowest in countries near the equator and the rate increases with latitude (47). In addition, there is accumulated data which indicates the enhanced risk of neurodevelopmental disabilities including ASD in children of immigrants to Europe and North America, mainly from Sub-Saharan Africa, East Africa, and Southeast Asia (48). Moreover, recent studies have revealed a link between month of conception and prevalence of ASD, with the highest rate among children conceived in the winter (49). Aside from maternal stress in the case of refugee immigrants, and seasonal pathogens, these observations were mainly attributed to maternal vitamin D deficiency (50). For example, autistic offspring of dark-skinned mothers who moved to higher latitudes and/or urban areas, resulting in lower level of maternal vitamin D due to decrease in sunlight exposure. Like vitamin D, melatonin production is highly related to sunlight exposure and varies seasonally. Therefore, we would like to propose that low or unbalanced levels of melatonin in the mother (20) or/and pineal/melatonin deficiency in the child, could provide an alternative or additional biological cause of vitamin D hypostasis (50, 51). Reexamination of the above observations focusing on the melatonin level may reveal new insights. For example, it may be that the seasonal conception effect (49) is due to the maternal melatonin level during gestation; not necessarily during the first trimester (15, 31), but rather in third trimester when maternal melatonin reaches its peak [summer, if conception took place in the winter (13)]. On the other hand, it could be that the effect is related to the date of birth. If this is the case, it can be assumed that the risk for developing ASD increases if a child has pineal dysfunction in addition to naturally low melatonin production over the summer months during a critical development period in the first years of life (52, 53). Additionally, breastfeeding was also examined, and proposed as a putative protective factor for ASD (54). Breastfeeding is the main source of melatonin during the first months of life (55), and therefore the seasonal effect on the infant might be the determining factor i.e., daylight hours. Though vitamin D and melatonin production are influenced by sunlight there are two significant parameters to be considered for melatonin alone. First, melatonin production significantly fluctuates both over the course of a day and seasonally (56). Second, melatonin synthesis is relatively sensitive to even dim afternoon light and is strongly influenced by artificial lighting, mainly in the blue light range. Russel et al. (57) proposed that night exposure to artificial light might be a risk factor for autism development. High levels of artificial lighting are common in the modern urban environment. Therefore, this factor could be related to the migration effect (48) when the frequency and intensity of night exposure to artificial lighting and hours of daylight change dramatically (56, 58). It is reasonable to assume that such a change will be most dramatic in populations where the biological clock and pineal function are inherently adjusted to very different illumination regimes. This could be comparable to studies on beta cell function in immigrants to Israel from Yemen; their insulin production was found to be insufficient for the new diet and lifestyle, causing high risk for type 2 diabetes (59). Moreover, the effect seems to peak when migration occurred around pregnancy and declined over time after immigration (60), which could indicate physiological adaptation of melatonin metabolism. For further possible effects of melatonin dysfunction on cognitive and behavioral aspects of ASD see the comprehensive review by Tordjman et al. (53).

Lastly, recent studies on Alzheimer's disease and general learning and memory research suggest that melatonin may also play an important role in synaptic stability, synaptic plasticity, and hippocampal neurogenesis through MT1 and MT2 receptors although, the mechanism is rather obscure (9, 61). As discussed in the next section, a defect in this function could also be linked to ASD symptoms.

## DMT and Neural Connectivity Abnormalities in Autism

**Figure 1:**
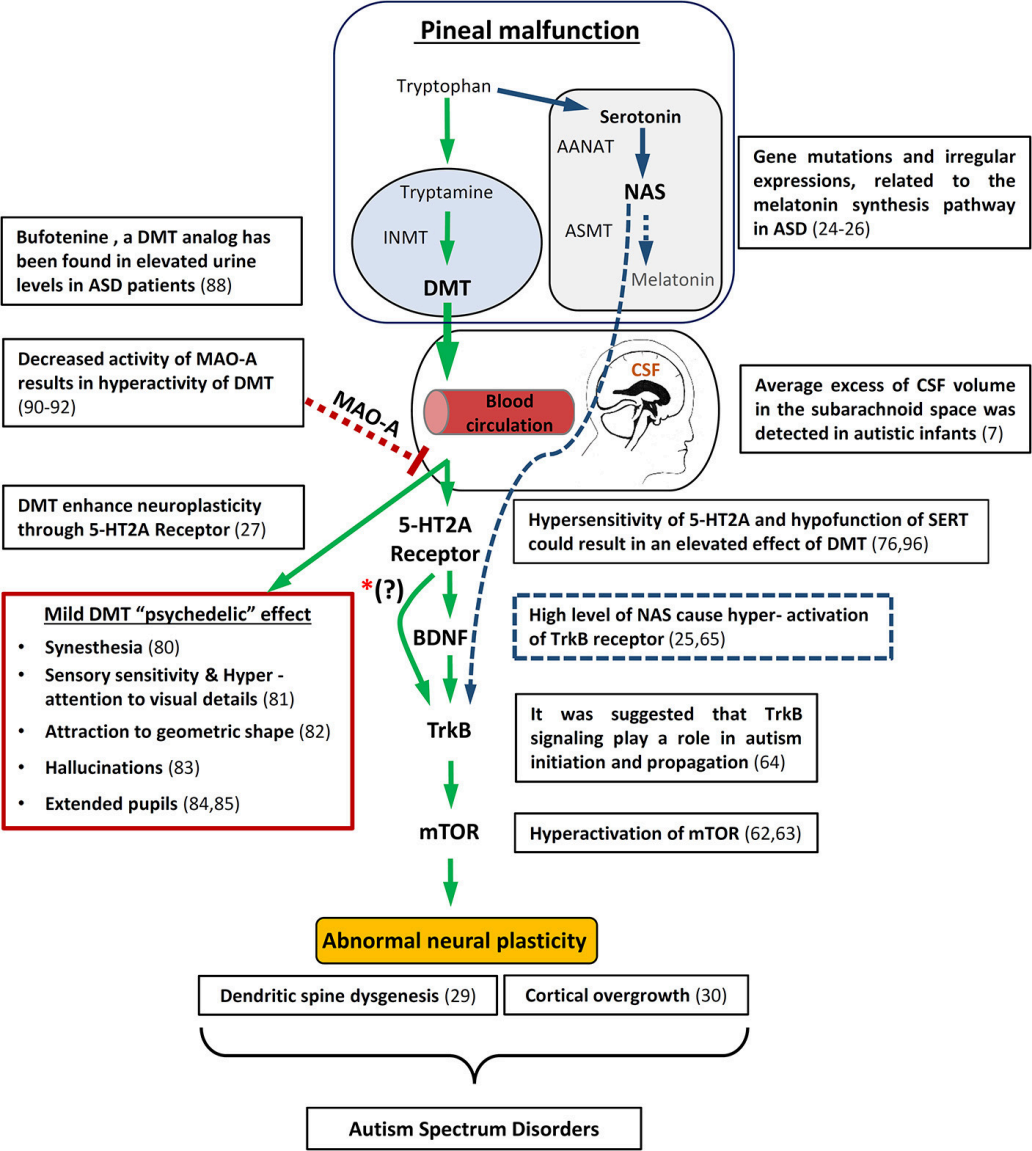
Schematic diagram illustrating how pineal gland dysfunction and/or MAO-A malfunction metabolism might result in hyperactivity of DMT, causing abnormal neuronal development and some of the behavioral/Physiological symptomns associated with ASD. On the upper right side is an overview of the way in which melatonin deficiency may also lead to abnormal neuronal plasticity through the melatonin precursor, NAS. In addition, relevant autism studies are specified. mTOR, mammalian target of rapamycin; BDNF, brain-derived neurotrophic factor; TrKB, tyrosine kinase B; INMT, Indolethylamine-N-mathyltransfersae; MAO-A, monoamine oxidase A; DMT, N,N-dimethyltryptamine; NAS, N-acetylserotonin; AANAT, Aralkylamine N-acetyltransferase; ASMT, Acetylserotonin O-methyltransferase; SERT, serotonin uptake transporter. ^*^(?) The interaction between 5-HT2A receptors and BDNF is still not entirely understood.

It has previously been suggested that abnormal neural plasticity such as cortical overgrowth (30) and dendritic spine dysgenesis (29) is a common neurological symptom in ASD. Abnormal activation of the mammalian target of rapamycin)mTOR) has been proposed as the cause for these abnormal developments in ASD (62, 63). In addition, it was suggested that disruption of brain-derived neurotrophic factor (BDNF) activity through tyrosine kinase B (TrkB) signaling might play a role in autism initiation and propagation (64). Interestingly, disruption of melatonin synthesis in ASD was associated with an increase of the melatonin precursors, serotonin and N-acetylserotonin (NAS). NAS was found to be an agonist of the TrkB receptor and therefore in conjunction with serotonin might contribute to the abnormal neural plasticity in ASD [Figure 1; (25, 65)].

A recent study by Ly et al. (27) showed that treatment of *Drosophila* and rats with DMT increases neuritogenesis, spinogenesis, and synaptogenesis through 5-HT2A receptor (5-HT2AR), TrkB, and mTOR signaling. The work of Ly et al. (27) is added to recent accumulated data on the effect of psychedelics (5-HT2AR agonists) like LSD and DMT structure analogs (66) on neuroplasticity, through cellular mechanisms such as the BDNF pathway, and even long term influence on gene expression in the brain (67, 68). The significant finding within the work of Ly et al. is that they show these effects for DMT–which was found to be endogenous in the brain. Based on the above, we propose that ASD may be affected by abnormal metabolism of endogenous DMT (Figure 1).

## The Endogenous DMT From the Pineal Gland

DMT is widely found in plants and animals, and although there have been indications for endogenous DMT in mammals since the 60 s, only in the last decade was it indisputably proven that DMT is present in humans (69). The source of endogenous DMT is unknown. One of the molecules being traced in order to identify the source of DMT is Indolethylamine-N-methyltransferase (INMT). INMT is thought to be the pivotal enzyme in the DMT production from the biogenic amine tryptamine, and therefore can indicate the sources of DMT. However, INMT methylates other substrates, and therefore its presence can only imply DMT production. INMT has been detected in many tissues in the body, primarily in the lungs, thyroid, and adrenal gland (70). Due to either popular trends or based on pure scientific reason, the pineal gland is the most studied area in the brain regarding DMT. The pineal gland was first proposed as the source of DMT in the brain since INMT had been found in human pineal, though its existence in other parts of the brain has been suggested as well (70–72). In 2013, Barker et al. (28, 73) provided the first strong indication for the existence of three endogenous forms of DMTs (DMT, 5-hydroxy-DMT and 5-methoxy-DMT) in the rat pineal. Still, the physiological function of DMT is unclear. Exogenous DMT has a clear and robust psychedelic effect, mainly through the activation of 5-HT2AR, and therefore it has been proposed that endogenous DMT might have a cognitive function. Yet, the role of DMT as an endogenous “psychedelic” remains controversial (74, 75). The main reason for the uncertainty is that only minute traces were detected (μg/kg), which were about 3 orders below the dose found to have psychedelic effects when delivered exogenously (mg/kg) (75, 76). It has even been considered a negligible non-functional concentration of byproduct from the tryptophan derivative indoleamines metabolism. However, there are reports (77) that link tumors in the pineal gland and peduncular hallucinosis (78) and florid psychotic symptoms (79). In connection to ASD, some autistic behavior such as synesthesia (80), sensory sensitivity, hyper attention to visual details (81), attraction to geometric shape (82), hallucinations (83), and extended pupils (84, 85) [note that this suppresses melatonin synthesis (86)] might be explained as a mild DMT psychedelic effect. Though, some of these symptoms such as synesthesia, might be the result of hyperconnectivity due to abnormal maturity of the brain (87), which as shown by Ly et al. (27) could also be a possible effect of DMT. **Importantly**, the DMT analog, 5-HO-DMT (Bufotenine) which is also a hallucinogen derived from tryptophan has been found in elevated urine levels in ASD patients (88).

## Is the Concentration of DMT in the Brain, Actually Higher Than What has Been Detected?

From the above, it is clear that any attempt to propose a biophysiological effect for DMT should first address the concentration issue. Barker (76) pointed out several critical factors that were not taken into account in the previous works, and might explain the tiny concentration of non-physiological endogenous DMT found in the brain. Three of these factors are relevant to pineal production of DMT, which as in the case of melatonin, might vary largely over the course of the day, seasonally and according to age (89). First, none of the works checked for circadian changes in DMT level. Second, the age of the examined subjects was not taken into consideration. Third, for obvious reasons, most data were collected from blood and urine samples and only a few tested DMT levels in the CSF, where the pineal gland directly deposits the compounds produced (in addition to the blood system). DMT concentration in the brain might also alter significantly according to the mental state of the subject, for example varying under stress conditions (89). Furthermore, although not directly related to the pineal gland, and as yet unproven, it was suggested that DMT might be accumulated in vesicles in the brain, building a reservoir–ready to release in effective concentrations (70). Another speculation is that DMT releases in a local and specific manner which allows it to reach an effective concentration. The small amount of detected concentration of DMT could be partly attributed to the efficient enzyme monoamine oxidase A (MAO-A), which readily catalyzes DMT (as well as other monoamines, such as serotonin, dopamine, and norepinephrine). However, there is evidence of decreased activity of MAO-A in the brain of children with ASD (90). Similar to other mutations associated with ASD, the MAO-A is an x-linked recessive gene and therefore correlates with the 4:1 male-to-female ratio. MAO-A dysfunction could also be caused by endogenous MAO-A inhibitors such as pinoline that is thought to be produced by the pineal gland (91). Moreover, Kałuzna-Czaplinska et al. (92) argued that their preliminary results indicate the existence of endogenous MAO-A inhibitor in the urine of ASD children, and that this abnormality is possibly related to pineal gland malfunction. Moreover, Davis et al. found that polymorphism of the MAO-A gene is associated with brain structure volumes in children with autism (93).

As for the work of Ly et al. (27) they tested concentrations of DMT that produced a psychedelic effect (10 mg/kg). However, they did not check lower concentrations, and therefore their finding could not rule out the possibility that neuroplasticity could be affected, over time, by the trace concentration. Interestingly, they did find the same effect on neural activity with 10 mg/kg (hallucinogenic dose) and 1 mg/kg (sub hallucinogenic dose). Alongside their finding regarding 5-HT2AR, it has been proposed that DMT and/or closely related compounds such as bufotenine, have an effect and possible physiological function in neural plasticity and immune system mechanisms through the activation of other receptors such as the sigma-1 (94) or other serotonin receptors (76). Yet, most of the known DMT receptors including the sigma-1 receptor, have lower affinity compared to 5-HT2AR (95).

Finally, It has been suggested that in ASD there is a decrease in serotonin binding to 5-HT2AR while hypersensitivity to agonist drugs (96), and hypo or hyper-function of the serotonin uptake transporter (SERT). DMT has been shown to be taken up into neuronal cells via SERT (76). Therefore, it is possible that hypo-function of SERT and/or hypersensitivity of 5-HT2AR could result in an elevated effect of DMT.

Drawing from all these recent studies, we postulate that pineal gland dysfunction and/or MAO-A malfunction metabolism, results in hyperactivity of DMT causing the abnormal neuronal development associated with ASD, possibly through the mechanism proposed by Ly et al. [Figure 1; (27)].

**In conclusion**, a wide range of recent studies provide new support for the hypothesis that pineal gland dysfunction and abnormal metabolism of its related products might be a significant, still unrevealed cause of ASD. Elevated levels of the endogenous psychotomimetic molecule bufotenine has been reported in urine of ASD patients (88), but as far as we know, no assessment of DMT in ASD patients has been performed. We hope that this perspective will encourage future research to test the hypothesis that DMT metabolism is associated with ASD development, and reexamination of the results attributed to vitamin D dysfunction; only this time, based on the pineal gland/melatonin malfunction premise. The results of such studies have the potential to lead to an effective and relatively simple treatment and maybe prevention procedure for ASD, using exogenous melatonin (97, 98) even for infants (99). In addition, treatment could include monitoring of light exposure in infants, toddlers, pregnant women, breastfeeding mothers, and providing pumped breastmilk to the infants according to the time it was expressed (day or night) in order to assure the proper levels of melatonin.

## Author Contributions

TS and NN conceived and drafted the manuscript.

### Conflict of Interest Statement

The authors declare that the research was conducted in the absence of any commercial or financial relationships that could be construed as a potential conflict of interest.
